# Supplementary material for: Stacking Model‐Based Classifiers for Dealing With Multiple Sets of Noisy Labels
Source: Biom J. 2025 Mar 12;67(2):e70042. doi: 10.1002/bimj.70042 (PMC11898607; doi:10.1002/bimj.70042)
Supplement: Supplementary file 1 — Supporting Information [file BIMJ-67-e70042-s001.zip › density-based-ensemble-model-main/competitors_real_data/netlab-master/netlab-master/help/glm.htm]

Netlab Reference Manual glm


# glm

## Purpose

Create a generalized linear model.

## Synopsis

```
net = glm(nin, nout, func)
net = glm(nin, nout, func, prior)
net = glm(nin, nout, func, prior, beta)
```

## Description

`net = glm(nin, nout, func)` takes the number of inputs
and outputs for a generalized linear model, together
with a string `func` which specifies the output unit activation function,
and returns a data structure `net`. The weights are drawn from a zero mean,
isotropic Gaussian, with variance scaled by the fan-in of the
output units. This makes use of the Matlab function
`randn` and so the seed for the random weight initialization can be
set using `randn('state', s)` where `s` is the seed value. The optional
argument `alpha` sets the inverse variance for the weight
initialization.

The fields in `net` are

```
  type = 'glm'
  nin = number of inputs
  nout = number of outputs
  nwts = total number of weights and biases
  actfn = string describing the output unit activation function:
      'linear'
      'logistic'
      'softmax'
  w1 = first-layer weight matrix
  b1 = first-layer bias vector
```

`net = glm(nin, nout, func, prior)`, in which `prior` is
a scalar, allows the field
`net.alpha` in the data structure `net` to be set, corresponding
to a zero-mean isotropic Gaussian prior with inverse variance with
value `prior`. Alternatively, `prior` can consist of a data
structure with fields `alpha` and `index`, allowing individual
Gaussian priors to be set over groups of weights in the network. Here
`alpha` is a column vector in which each element corresponds to a
separate group of weights, which need not be mutually exclusive. The
membership of the groups is defined by the matrix `index` in which
the columns correspond to the elements of `alpha`. Each column has
one element for each weight in the matrix, in the order defined by the
function `glmpak`, and each element is 1 or 0 according to whether
the weight is a member of the corresponding group or not.

`net = glm(nin, nout, func, prior, beta)` also sets the
additional field `net.beta` in the data structure `net`, where
beta corresponds to the inverse noise variance.

## See Also

`glmpak`, `glmunpak`, `glmfwd`, `glmerr`, `glmgrad`, `glmtrain`

---

**Pages:**
Index


---

Copyright (c) Ian T Nabney (1996-9)
